# Immunological profile in cerebrospinal fluid of patients with multiple sclerosis after treatment switch to rituximab and compared with healthy controls

**DOI:** 10.1371/journal.pone.0192516

**Published:** 2018-02-08

**Authors:** Pierre de Flon, Lars Söderström, Katarina Laurell, Ann Dring, Peter Sundström, Martin Gunnarsson, Anders Svenningsson

**Affiliations:** 1 Dept of Pharmacology and Clinical Neuroscience, Neurology, Umeå University, Umeå, Sweden; 2 Unit of Research, Education and Development, Östersund Hospital, Region Jämtland Härjedalen, Östersund, Sweden; 3 Dept of Neurology, Faculty of Medicine and Health, Örebro University, Örebro, Sweden; 4 Dept of Clinical Sciences, Danderyd Hospital, Karolinska Institutet, Stockholm, Sweden; University of Münster, GERMANY

## Abstract

**Objective:**

To investigate changes in the cerebrospinal fluid (CSF) immunological profile after treatment switch from first-line injectables to rituximab in patients with relapsing-remitting MS (RRMS), and to compare the profile in MS patients with healthy controls (HC).

**Method:**

Cerebrospinal fluid from 70 patients with clinically stable RRMS and 55 HC was analysed by a multiplex electrochemiluminescence method for a broad panel of cytokines and immunoactive substances before, and over a two-year period after, treatment switch to rituximab. After quality assessment of data, using a predefined algorithm, 14 analytes were included in the final analysis.

**Results:**

Ten of the 14 analytes differed significantly in MS patients compared with HC at baseline. Levels of IP-10 (CXCL10), IL-12/23p40, IL-6, sVCAM1, IL-15, sICAM1 and IL-8 (CXCL8) decreased significantly after treatment switch to rituximab. The cytokines IP-10 and IL-12/IL-23p40 displayed the largest difference versus HC at baseline and also the largest relative reduction after therapy switch to rituximab.

**Conclusion:**

We found significant changes in the immunological profile after therapy switch to rituximab in RRMS in the direction towards the values of HC. IP-10 and IL12/IL-23p40 deserve further studies as part of the immunopathogenesis of MS as well as for the mode of action of rituximab in MS.

## Introduction

Multiple sclerosis (MS) is an inflammatory disease of the central nervous system (CNS) where the main feature is an autoimmune attack on CNS myelin leading to damage of the myelin sheath and, if not treated adequately, a progressive loss of axons and subsequent irreversible disability [1,2]. The mechanisms inducing the inflammatory response in MS are still under intense investigation. The earlier predominant view that the inflammatory activity is mainly dependent on pro-inflammatory T-cells has been challenged by the results of treatment with B-cell depleting agents. The effect of B-cell depletion on the inflammatory activity in MS has been confirmed in several trials [3–6]. The putative biological role of B-cells in MS may be to regulate tolerance and autoimmunity through antigen-presenting characteristics and involvement in cytokine networks [7,8].

The development of multiplex technology, simultaneously measuring multiple analytes, provides a tool for analysing large panels of different substances from small volume samples. Such studies can provide new perspectives on the mechanisms involved in the pathogenesis of MS and the mode of action of novel disease modifying therapies. Reported cytokine levels in cerebrospinal fluid (CSF) in various diseases, including MS, are diverse and comparison between different studies is complicated by heterogeneity in terms of clinical groups and methodology [9].

Few studies have explored the changes in cytokine levels in CSF in relation to rituximab treatment in MS. A significant reduction of the level of B-cell activating factor (BAFF) was described after intrathecal administration of rituximab in nine patients of which four with relapsing-remitting MS (RRMS) and five with secondary progressive MS (SPMS) [10]. Further, in a single-case study on SPMS, changes of a broad panel of cytokines were reported after repeated intrathecal administrations of rituximab [11]. To our knowledge, only one study has addressed the changes in immunological profile in the CSF of RRMS patients after intravenous (iv) administration of rituximab [12], with a reduction of CXCL13 and CCL19 at 24 weeks after add-on treatment with rituximab.

We have previously reported the results of a phase II trial (the STRIX trial: Switch-To-RItuXimab in MS) evaluating the inflammatory activity in patients with clinically stable RRMS after a therapy switch from the first-line injectables interferon (IFN) -beta or glatirameracetate (GA) to rituximab [13]. The aim of the present study was to explore and describe the change of the immunological profile in the CSF in this patient population over the two-year study period and in comparison with healthy controls (HC). In order to avoid spurious interpretations, a systematic quality assessment algorithm was applied.

## Method

### Study participants

**Fig 1:**
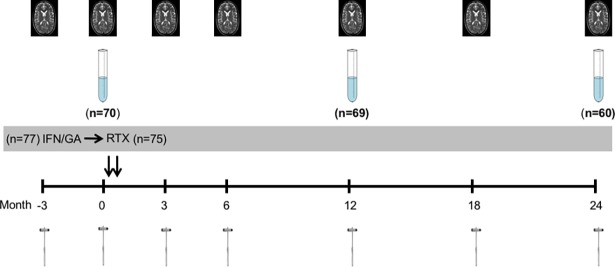
Study design of the STRIX-MS study. Sample tubes indicate the timing of LPs. The reflex hammers indicate the timing of clinical assessments and the MRI pictures indicate the timing of radiological assessments. IFN- interferon beta, GA- glatirameracetate, RTX- rituximab.

The study population was recruited from the 77 patients with clinically stable RRMS included in the STRIX-MS trial (Fig 1) (13). From the original 77 patients, two withdrew consent and two declined lumbar puncture (LP) before treatment switch. For the purpose of this study another three patients were excluded due to confounding factors (one re-diagnosed as CADASIL after closure of the STRIX-MS trial, one with a ventriculo-peritoneal shunt and one receiving natalizumab as rescue treatment during the first year due to therapy failure). The remaining 70 patients were included in this study; 60 being treated with IFN-beta and 10 patients with GA at the time of inclusion. During the follow-up one patient declined LP at year one and two and another six patients at year two. Three patients received rescue therapy during the second year according to the predefined study criteria and were excluded from the analysis at year two. The numbers of patients available for analysis at each time-point are described in Fig 1. The blinded analysis of the MRI after the closing of the STRIX-trial revealed MRI activity in 14 patients prior to the therapy switch. Three of these patients together with another four patients showed signs of inflammatory activity on MRI at some point during the follow-up after the therapy switch.

The healthy controls (HC) were volunteers without diagnosis of neurological disease and without first-degree relatives with such disease. Controls were recruited from the Umeå area through advertisement in local newspapers and posters. An experienced research nurse performed screening for inclusion and exclusion criteria and 55 HC were included. There were statistically significant differences in respect of sex and age between the HC and MS patients (Table 1).

**Table 1:** Demographics for MS patients and healthy controls.

Demographics	MS-patients		Healthy controls		p-value
**Number of subjects**	70		55		
**Women (%)**	48	(68)	28	(51)	p = 0.045^a^
**Age at inclusion in years,** **mean (SD)**	41.3	(7.9)	37.6	(13.0)	p = 0.036^b^
**Duration of disease in years,** **mean (SD)**	9.6	(6.9)			
**Duration of treatment in months,** **mean (SD)**	63.1	(39.1)			
**EDSS at inclusion,** **median (range)**	1.5	(0–5)			

SD- standard deviation.

^a^ calculated by Pearson Chi-square test

^b^ calculated by Mann-Whitney test

### Study drug

Rituximab (Mabthera^®^, Roche) was given iv as two doses of 1000 mg two weeks apart. The injection therapy (IFN-beta or GA) was discontinued at the time of the first infusion.

### CSF collection

Lumbar puncture was performed before treatment switch and at months 12 and 24. Cerebrospinal fluid was collected in 10 ml polypropylene tubes (Sarstedt) and centrifuged at 400g for 10 minutes. The supernatant was pipetted off and dispensed in 9 fractions of 1 ml in 1.5 ml polypropylene tubes (Sarstedt) and stored at -80°C.

### Multiplex cytokine assay

The MesoScale Discovery V-PLEX^®^ multiplex electrochemiluminescence assay platform (MSD; MesoScale Discovery, Rockville, MD, USA) was used to profile CSF samples for immunoactive components according to the manufacturer’s instructions. Briefly, analytes in the CSF were bound by primary capture antibodies located on specified carbon spots within a 96-well plate format (up to 10 spots/well). Calibrators were prepared to yield a 7- or 8-point standard curve and blank. Directions for the dilution of CSF specifically were not provided so samples were diluted as recommended for serum and plasma. All samples and calibrators were assayed in duplicate wells. Electrical stimulation of each spot in turn caused the emission of light from bound SULFO-TAG labelled detection antibodies. The signals were acquired using a Sector Imager 2400 with Discovery Workbench software v.3.0 and converted to concentrations using standard curves.

The patient samples were analysed in two different batches. Thirty-six patients that had completed the two-year follow-up in the STRIX-trial by the summer of 2014 were analysed in the first set of experiments (batch 1) using the full MSD V-PLEX^®^ Neuroinflammation Human Panel 1 (HP1), comprising 36 analytes arrayed across six 96-well MSD plates. Data from batch 1 underwent preliminary analysis. We determined that further analyses in the remaining patients were meaningful only for analytes fulfilling the following three criteria: 1) > 50% of the values above the detection limit, 2) >50% of the values with a CV <25% and 3) a statistically significant difference detectable after treatment switch. Twenty-two analytes fulfilled these criteria and were thus analysed in the CSF from the remaining patients in the study (n = 34) in a second set of experiments (batch 2). In batch 2 the analytes were arranged in a custom panel using the same antibodies and technical properties as HP1, arrayed across five different 96-well plates (S1 Table). The results of batch 2 were pooled with the results of the corresponding analytes from batch 1 for the final statistical analyses. The samples from the HC were analysed in both batches for inter-batch quality control.

The specific plate layouts were designed for each batch. In order to ensure a balanced set, RRMS cases and HC were included on each plate, along with two inter-assay control samples (QC), and all time points (month 0, 12, 24) from each patient were together on the same plate. Each QC sample was created by combining equal volumes of CSF from six patients and then frozen as single use aliquots to be included on each plate in the respective batches.

### Quality control assessment

#### Quality control of standard curve and definition of lowest level of quantification

For each analyte, the coefficient of variation (CV) was calculated from the duplicate calibrators establishing the standard curves. The CV was <25% in the middle and higher range of all analytes, but consistently >25% in the lower range (S2 Table). This was higher than expected from the certificates of analysis from the manufacturers. We therefore defined the lowest level of quantification (LLoQ) as 85% of the lowest value on the calibrator curve with a CV <25%. All values below the LLoQ were replaced by half the value of LLoQ for statistical analysis.

Analytes with >50% of results <LLoQ were excluded from further analysis (Table 2).

**Table 2:** Lowest level of quantification and results of quality assessment.

					Median (Q_1_-Q_3_)						* *
Analyte	LLoQ	Healthy control	(n = 55)		Month 0	(n = 70)	Month 12	(n = 69)	Month 24	(n = 60)	Outcome of quality assesment
*IFN-γ*	*1*.*14*	*-*			*-*		*-*		*-*		*Excluded due to >50% of values <LLoQ*
*IL-10*	*0*.*16*	*-*			*-*		*-*		*-*		
*TNF-α*	*0*.*42*	*-*			*-*		*-*		*-*		
*MCP-4 (CCL13)*	*5*.*17*	*-*			*-*		*-*		*-*		
*Tie-2*	*65*.*6*	*-*			*-*		*-*		*-*		
*MDC (CCL22)*	*8*.*14*	*-*			*-*		*-*		*-*		*Excluded due to >25% of values with CV >25% in duplicates*.
*TARC (CCL17)*	*2*.*98*	*-*			*-*		*-*		*-*		
*MIP-1α (CCL3)*	*3*.*35*	*-*			*-*		*-*		*-*		
IP-10 (CXCL10)	0.52	284	(211–423)		938	(665–1650)	616	(434–841)	597	(432–978)	Accepted for final statistical calculations.
IL-12/IL-23p40	2.31	3.77	(3.38–4.67)		7.23	(5.51–13.2)	5.19	(3.63–6.97)	6.29	(4.16–10.7)	
IL-6	0.59	1.11	(0.89–1.50)		1.42	(1.08–1.78)	1.21	(0.93–1.50)	1.23	(0.94–1.65)	
sVCAM-1	14.0	6150	(5300–7860)		8070	(6770–9760))	7350	(6400–8500)	7580	(6380–8830)	
IL-15	0.14	2.27	(1.93–2.57)		2.49	(2.15–3.10)	2.28	(1.95–2.80)	2.42	(2.01–2.87)	
sICAM-1	13.9	1630	(1400–2050)		1980	(1690–2480)	1830	(1570–2280)	1980	(1580–2410)	
IL-8 (CXCL8)	0.42	34.1	(29.4–38.2)		44.0	(37.1–51.4)	41.7	(34.7–49.2)	42.1	(34.5–48.5)	
VEGFD	4.74	41.0	(28.1–54.7)		41.4	(33.7–50.1)	44.6	(35.2–54.5)	45.8	(34.3–57.7)	
IL-7	0.53	1.35	(1.12–1.55)		1.10	(0.92–1.38)	1.22	(0.99–1.55)	1.13	(0.87–1.34)	
IL-5	0.17	0.57	(0.48–0.77)		0.51	(0.38–0.65)	0.52	(0.42–0.64)	0.53	(0.44–0.65)	
MCP-1 (CCL2)	0.40	317	(261–364)		327	(268–399)	316	(262–368)	316	(269–379)	
MIP-1β (CCL4)	3.44	10.5	(8.63–14.0)		15.5	(12.1–19.9)	14.2	(11.0–20.4)	14.1	(11.4–19.5)	
CRP	1.99	1360	(704–2380)		2450	(1330–6200)	2240	(1340–5720)	2290	(1170–4740)	
SAA	64.5	667	(517–938)		944	(672–1420)	814	(591–1410)	906	(543–1470)	

The table presents the lowest level of quantification (LLoQ) and reasons for exclusion after data quality assessment on the pooled results from batches 1 and 2. All values are in pg/mL. The eight analytes excluded according to the final quality control assessment of the pooled results in the study are written in italics and the reason for exclusion noted in the column “Outcome of quality assessment”. The total number of patients included at each time point and the number of Healthy Controls are presented in brackets in the heading of the table.

#### Intra-assay accuracy for individual samples

The results for each of the remaining analytes were assessed regarding CV for each individual pair of duplicates. Samples with CV >25% were excluded. Analytes with <75% of the samples remaining were excluded from further statistical analysis (Table 2).

#### Inter-assay accuracy

Analytes passing the quality assessment described above were checked for inter-plate variability by calculating the CV for the two QC samples included on each plate. All CV were <25% and considered acceptable.

### Statistical analysis

All results are presented as median with interquartile range (IQR). Statistical differences in demographic parameters were tested by Chi-square test or Fisher exact test for sex and Mann-Whitney test for age. The level of statistical significance between the results of MS patients at different time-points was tested by Wilcoxon signed-rank test and the difference between the study population at the different time points and HC was tested using the Kruskal Wallis rank test. In order to compensate for multiple comparisons, the level of significance was adjusted according to Holm-Bonferroni. All data handling and statistical analyses were made using SAS 9.4 (SAS Institute Inc, Cary, NC, USA) and Matlab R2016 (MathWorks Inc, USA).

### Ethics and regulatory statement

This study was approved by the Ethics Committee in Umeå (Dnr 2010-315-31M, Dnr 2011-39-31M and Dnr 2017-37-32M) and the main study, STRIX, was registered in the EU Clinical Trial Register (EudraCT no 2010-023012-38). Written informed consent was obtained from each patient and healthy control.

## Results

### Comparison of immunological profile before and after treatment switch to rituximab

**Fig 2:**
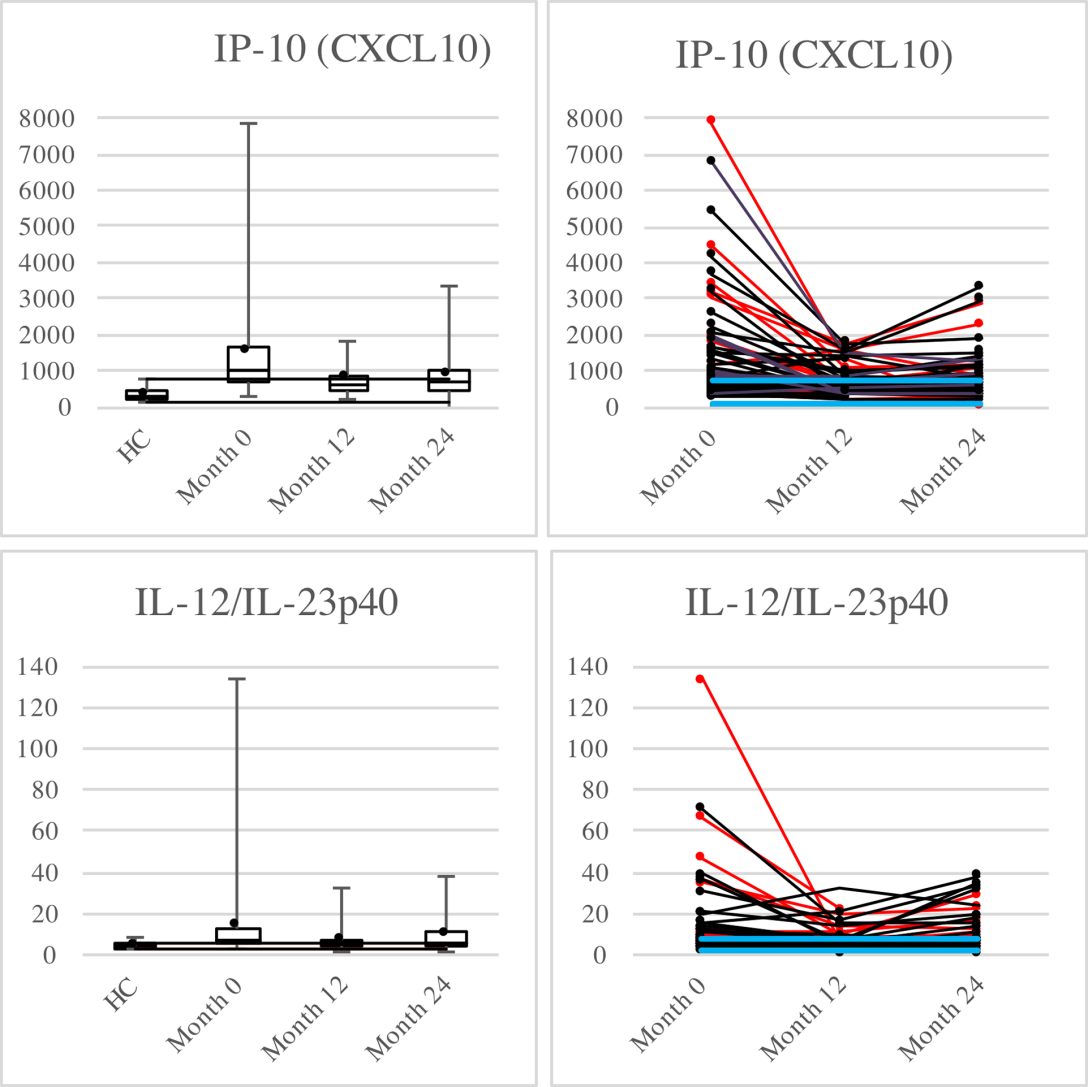
Box-plots and dot-line diagram for IP-10 and IL-12/IL-23p40. All values in pg/mL. In the box-plots the whiskers represent the min/max respectively, dots represent the mean and the black lines mark the level of the lowest/highest value within Q1-1.5xIQR/Q3+1.5xIQR respectively. The dot-line diagrams display the values for each MS patient. HC are not included. Patients displaying subclinical inflammatory activity on MRI at any time point during the STRIX-study are marked by red lines and dots, patients without radiological activity are marked by black. The green lines represent LLoQ and the blue lines min/max value respectively for HC.

Of the 14 analytes that fulfilled the quality requirements (Table 2) the median level one year after therapy switch to rituximab was significantly reduced for IP10, IL-12/23p40, IL-6, sVCAM-1, IL-15, sICAM-1 and IL-8 (Table 3). The relative differences were greatest for IP-10 (34%) and IL-12/23p40 (28%). These data are presented in more detail in Fig 2. Graphics for the remainder of the analytes are available as supplemental material (S1 Fig).

**Table 3:** Changes in immunological profile after therapy switch to rituximab.

Analyte	Month 0	Month 12 vs month 0				Month 24 vs month 0			
	Median	Median	Relative change (%)	p-value	N	Median	Relative change (%)	p-value	N
IP-10 (CXCL10)	938	616.0	-34%	**<0.0001**	64	597	-36%	**<0.0001**	53
IL-12/IL-23p40	7.23	5.19	-28%	**<0.0001**	56	6.29	-13%	0.0050	47
IL-6	1.42	1.21	-15%	**0.0012**	52	1.23	-13%	0.2006	43
sVCAM1	8070	7350	-9%	**<0.0001**	69	7580	-6%	**0.0015**	60
IL-15	2.49	2.28	-8%	**<0.0001**	69	2.42	-3%	**<0.0001**	60
sICAM1	1980	1830	-7%	**0.0001**	69	1980	0%	0.4572	60
IL-8 (CXCL8)	44.0	41.7	-5%	**0.0013**	68	42.1	-4%	0.0045	59
VEGFD	41.4	44.6	8%	**0.0011**	67	45.8	11%	0.0066	60
IL-7	1.10	1.22	11%	0.0065	48	1.13	3%	0.5610	37
IL-5	0.51	0.52	2%	0.9030	51	0.53	5%	0.1048	44
MCP-1 (CCL2)	327	316	-3%	0.0086	66	316	-3%	0.3429	58
MIP-1β (CCL4)	15.5	14.2	-8%	0.1917	46	14.1	-9%	0.7653	37
CRP	2450	2240	-8%	0.5154	63	2290	-6%	0.5922	53
SAA	944	814	-14%	0.0243	61	906	-4%	0.2088	55

All values in pg/mL. Relative changes are given as percent of median at month 12 and month 24 respectively compared to month 0. N = the number of samples available for paired statistical analysis. Changes reaching statistical significance after correction for 28 multiple comparisons according to Holm-Bonferroni are indicated in bold.

### Immunological profile in MS patients compared with healthy controls

While still on injectable treatment (IFN-beta or GA), the median levels of IP-10 (CXCL10), IL-12/23p40, sVCAM-1, IL-8 (CXCL8), MIP-1β (CCL4), CRP, IL-15, sICAM-1 and SAA were significantly higher for MS patients compared with HC. In contrast, the median level of IL-7 was significantly lower in MS patients. Also in this aspect, the differences were most prominent for IP-10 and IL-12/23p40 (Fig 2). A summary of the relation between HC and MS patients at the various time points after treatment switch is presented in supplemental material (S3 Table).

## Discussion

In this study, we report changes in the immunological profile of CSF from patients with clinically stable RRMS following therapy switch from first-line injectables to rituximab. The two cytokines displaying the most prominent relative changes after treatment switch to rituximab were IP-10 and IL-12/23p40. The level of these two cytokines were also the most elevated in RRMS when compared to HC, making them particularly interesting as possible mediators of a beneficial treatment effect from rituximab in MS.

IP-10 (CXCL10) is a small protein described as an “inflammatory chemokine” crucial to leukocyte trafficking as well as the perpetuation of inflammation in MS and various other autoimmune diseases [9, 14, 15]. In a previous study, the level of IP-10 in CSF was reduced after initiation of natalizumab treatment in MS [16]. Our results are consistent with this finding and further suggest that B-cell depleting therapy may have similar effects to natalizumab on IP-10 levels. Since the receptor for IP-10, CXCR3, is preferentially expressed on activated Th1 cells [17] our results imply a possible mechanism whereby B-cell depletion may indirectly affect T-cell function.

Another possible pathway for the indirect effect of rituximab on T-cells was indicated through the finding of a reduced level of IL-12/IL-23p40 following treatment switch, as IL-12-induced Th1 expansion is thought to play an important role in MS inflammation. The p40 subunit is common to IL-12 and IL-23 with a role in MS yet to be clarified. It has been demonstrated that p40-deficient mice are resistant to experimental autoimmune encephalitis (EAE) [18]. However, a study of the p40-blocking monoclonal antibody ustekinumab did not have any effect on the inflammatory activity measured by MRI in MS [19] demonstrating the difficulty in interpreting the function of a single cytokine in a large immunological network.

Both in the case of IP-10 and IL-12/IL-23p40, the decrease at month 12 seen in individual patients with high values at treatment shift tended to be followed by an increase at month 24, which is in agreement with our previous findings with return of inflammatory activity seen on MRI and by Neurofilament-Light protein in some patients after month 12 [13]. This observation opens up a possibility of using these two cytokines as markers for disease activity and as indicators of persistent treatment effect by rituximab.

There are several limitations in our study. With the patient population, by necessity, already on treatment when starting rituximab therapy, we can only speculate how our findings may relate to patients naïve to immunomodulating treatments. However, through comparison with a group of healthy individuals our results may be related to normal physiology. Furthermore, the introduction of rituximab at the time of withdrawal of the injectable therapy makes it impossible to exclude that parts of the observed effects were attributed to the withdrawal of IFN-beta or GA. The inclusion of patients with a clinically stable disease, according to the inclusion criteria of the STRIX-trial, reduces the possibility to explore changes related to an uncontrolled active disease and the limited sample size does not make it possible to perform any subgroup analysis. There was a statistically significant difference between the MS-patients and the HC regarding sex and age. It has been shown previously that these parameters might affect the levels of at least some cytokines in healthy individuals [20] as well as in RRMS [21] but not for any of the cytokines included in our final results. The difference in mean age was in a range that any major impact on the conclusions is unlikely. The MSD assay had not been developed specifically for use with CSF and appropriate dilution factors were assumed to be similar to those for other biological fluids. Since the prepared standard curves displayed a lower precision in the lower ranges than expected from the certificates of analysis provided by the manufacturer it is likely that the dynamic range of some of the assays was sub-optimal for CSF, as prepared in this study. We applied a systematic quality assessment strategy to exclude analytes for which good data were not attainable.

This study failed to provide assessable results for some analytes of great interest in the cytokine network involved in MS. One of them, IL-17, well described as an important key player in several neuroimmune interactions [22], was not detectable in a reliable manner in batch 1 and therefore excluded from the final analysis. In the pooled data interferon-γ did not reach detectable limits which is in accordance with some previous studies [16, 23]. IL-10, implicated to have an important role in the immunoregulatory function of B-cells, was likewise not reliably detectable. The difficulties in obtaining detectable levels for these analytes are described in previous studies [9, 23]. Another cytokine recently shown to be of interest in MS pathophysiology, CXCL13 [12], was unfortunately not part of the MSD panel selected for this study. Further studies of the present material could be justified specifically addressing this cytokine.

In summary, we observed significant and persistent changes in the CSF immunological profile after a switch to rituximab treatment in clinically stable patients with RRMS. The observed changes were in the direction of normalisation and add to the growing information on possible mechanisms behind B-cell depleting therapy in MS. The two cytokines IP-10 and IL-12/IL-23p40 merit further studies regarding both the pathophysiology of MS and as markers for rituximab treatment effect.

## Supporting information

S1 Table Overview of analytes included in batch 1 and 2 respectively. (DOCX)

S2 Table CV values for calibrators in batch 1 and 2 used for calculation of LLoQ. (DOCX)

S3 Table A summary of the relation between HC and MS patients at the various time points. All values in pg/mL. Differences at various time-points versus HC reaching statistical significance after correction for 42 multiple comparisons according to Holm-Bonferroni are indicated in bold. **N = number of samples accepted for statistical analysis.
(DOCX)

S1 Fig Boxplots and dot-line diagrams for the analytes not presented in the main manuscript. All values in pg/mL. The fence of the whiskers represents the max-min values. The green lines represent the LLoQ, the blue lines represent the min-max of the HC.
(PDF)
