# Supplementary material for: Immunological profile in cerebrospinal fluid of patients with multiple sclerosis after treatment switch to rituximab and compared with healthy controls
Source: PLoS One. 2018 Feb 8;13(2):e0192516. doi: 10.1371/journal.pone.0192516 (PMC5805315; doi:10.1371/journal.pone.0192516)
Supplement: S1 Table — (DOCX) [file pone.0192516.s001.docx]

| MSD Panel | Analytes included batch 1 | Analytes included batch 2 |
| --- | --- | --- |
| Proinflammatory Panel 1 | IL-1β, IL-2, IL-4, IL-6, IL-8, IL-10, IL-13, IFN-γ, TNF-α | IL-6, IL-8, IL-10, IFN-γ, TNF-α |
| Cytokine Panel 1 | IL-1α, IL-5, IL-7,  IL-12/23p40, IL-15, IL-16, TNF-β, VEGF | IL-5, IL-7, IL12/23p40,  IL-15 |
| Chemokine Panel 1 | Eotaxin (CCL11),  Eotaxin 3(CCL26),  IP10 (CXCL10), MCP1(CCL2),  MCP4 (CCL13),  MDC (CCL22),  MIP1α (CCL3),  MIP1β (CCL4),  TARC (CCL17) | IP10 (CXCL10), MCP1(CCL2),  MCP4 (CCL13),  MDC (CCL22),  MIP1α (CCL3),  MIP1β (CCL4),  TARC (CCL17) |
| Angiogenesis Panel 1 | BFGF, FLT1, TIE2, VEGF-C, VEGF-D | Tie2, VEGF-D |
| Vascular injury Panel 2 | CRP, SAA, sICAM1, sVCAM1 | CRP, SAA, sICAM1, sVCAM1 |
| IL-17A Panel | IL-17A |  |
