# Supplementary material for: Immunological profile in cerebrospinal fluid of patients with multiple sclerosis after treatment switch to rituximab and compared with healthy controls
Source: PLoS One. 2018 Feb 8;13(2):e0192516. doi: 10.1371/journal.pone.0192516 (PMC5805315; doi:10.1371/journal.pone.0192516)
Supplement: S2 Table — (DOCX) [file pone.0192516.s002.docx]

|  |  | **Batch 1** | | | | | |  | **Batch 2** | | | | | |  |
| --- | --- | --- | --- | --- | --- | --- | --- | --- | --- | --- | --- | --- | --- | --- | --- |
|  |  | **Defined** | **Total no.** | **Total** | **CV %** |  |  |  | **Defined** | **Total no.** | **Total** | **CV %** |  |  |  |
| **Panel** | **Analyte** | **Conc.** | **of obs.** | **mean** | **All** | **Inter** | **Intra** |  | **Conc.** | **of obs.** | **mean** | **All** | **Inter** | **Intra** |  |
| 1 | IFN-γ | 0 | 6 | 0.1485 | 56.47 | 53.33 | 17.29 |  | 0 | 5 | 0.1094 | 63.36 | 63.05 | 6.30 |  |
|  |  |  |  |  |  |  |  |  | 0.081787 | 8 | 0.1323 | 57.59 | 55.55 | 26.43 |  |
|  |  | 0.344238 | 8 | 0.3433 | 37.87 | 0.00 | 37.87 |  | 0.327148 | 10 | 0.3504 | 86.37 | 0.00 | 86.37 |  |
|  |  | 1.376953 | 10 | 1.5308 | 8.01 | 0.00 | 8.01 |  | 1.308593 | 10 | 1.4197 | 7.32 | 2.35 | 6.98 |  |
|  |  | 5.507813 | 10 | 5.4559 | 3.95 | 0.86 | 3.87 |  | 5.234375 | 10 | 4.9976 | 6.32 | 0.00 | 6.32 |  |
|  |  | 22.03125 | 10 | 21.8819 | 3.17 | 0.37 | 3.15 |  | 20.9375 | 10 | 20.9894 | 2.00 | 0.00 | 2.00 |  |
|  |  | 88.125 | 10 | 88.9641 | 2.89 | 0.00 | 2.89 |  | 83.75 | 10 | 83.5342 | 4.14 | 0.00 | 4.14 |  |
|  |  | 352.5 | 10 | 350.0971 | 2.77 | 0.85 | 2.65 |  | 335 | 10 | 338.7084 | 5.59 | 0.00 | 5.59 |  |
|  |  | 1410 | 10 | 1412.6935 | 1.29 | 0.00 | 1.29 |  | 1340 | 4 | 1342.9215 | 2.07 | 0.00 | 2.07 |  |
| 1 | IL-10 | 0 | 5 | 0.0124 | 123.30 |  |  |  | 0 | 4 | 0.0227 | 81.96 | 81.55 | 8.16 |  |
|  |  |  |  |  |  |  |  |  | 0.018372 | 8 | 0.0230 | 74.96 | 57.03 | 50.74 |  |
|  |  | 0.079102 | 10 | 0.0895 | 17.14 | 0.00 | 17.14 |  | 0.073486 | 10 | 0.0851 | 32.57 | 0.00 | 32.57 |  |
|  |  | 0.316406 | 10 | 0.3084 | 4.24 | 0.00 | 4.24 |  | 0.293945 | 10 | 0.2959 | 8.24 | 2.23 | 7.97 |  |
|  |  | 1.265625 | 10 | 1.2644 | 3.40 | 2.53 | 2.42 |  | 1.175781 | 10 | 1.1375 | 4.11 | 2.73 | 3.20 |  |
|  |  | 5.0625 | 10 | 4.9076 | 5.27 | 0.00 | 5.27 |  | 4.703125 | 10 | 4.6661 | 3.63 | 0.00 | 3.63 |  |
|  |  | 20.25 | 10 | 20.5952 | 2.77 | 1.64 | 2.29 |  | 18.8125 | 10 | 18.8430 | 3.89 | 0.00 | 3.89 |  |
|  |  | 81 | 10 | 82.4479 | 5.13 | 0.00 | 5.13 |  | 75.25 | 10 | 76.7795 | 3.75 | 0.00 | 3.75 |  |
|  |  | 324 | 10 | 322.4518 | 2.23 | 0.00 | 2.23 |  | 301 | 4 | 299.7834 | 2.01 | 0.00 | 2.01 |  |
| 1 | IL-6 | 0 | 2 | 0.0987 | 34.43 | 34.26 | 3.43 |  | 0 | 4 | 0.0522 | 81.39 | 80.99 | 8.10 |  |
|  |  |  |  |  |  |  |  |  | 0.039673 | 9 | 0.0635 | 59.03 | 62.99 | 19.80 |  |
|  |  | 0.187744 | 10 | 0.2602 | 59.10 | 0.00 | 59.10 |  | 0.158691 | 10 | 0.2228 | 55.88 | 0.00 | 55.88 |  |
|  |  | 0.750977 | 10 | 0.9405 | 15.59 | 0.00 | 15.59 |  | 0.634766 | 10 | 0.7111 | 6.36 | 5.50 | 3.68 |  |
|  |  | 3.003906 | 10 | 3.1952 | 4.92 | 2.54 | 4.30 |  | 2.539063 | 10 | 2.4606 | 6.54 | 0.00 | 6.54 |  |
|  |  | 12.01563 | 10 | 10.8792 | 6.57 | 0.00 | 6.57 |  | 10.15625 | 10 | 9.5277 | 3.99 | 3.06 | 2.76 |  |
|  |  | 48.0625 | 10 | 44.2612 | 4.57 | 0.00 | 4.57 |  | 40.625 | 10 | 37.6920 | 8.25 | 0.00 | 8.25 |  |
|  |  | 192.25 | 10 | 198.6684 | 4.13 | 0.00 | 4.13 |  | 162.5 | 10 | 178.1810 | 9.72 | 6.17 | 7.79 |  |
|  |  | 769 | 10 | 841.3932 | 5.81 | 4.65 | 3.81 |  | 650 | 4 | 724.5356 | 8.23 | 0.00 | 8.23 |  |
| 1 | IL-8 | 0 | 4 | 0.0166 | 76.17 | 75.80 | 7.58 |  | 0 | 6 | 0.0188 | 47.33 | 0.00 | 47.33 |  |
|  |  |  |  |  |  |  |  |  | 0.030823 | 8 | 0.0396 | 68.55 | 33.37 | 60.70 |  |
|  |  | 0.123779 | 10 | 0.1335 | 28.02 | 0.00 | 28.02 |  | 0.123291 | 10 | 0.1650 | 63.51 | 0.00 | 63.51 |  |
|  |  | 0.495117 | 10 | 0.5370 | 6.01 | 0.00 | 6.01 |  | 0.493164 | 10 | 0.5064 | 7.12 | 1.07 | 7.04 |  |
|  |  | 1.980469 | 10 | 1.9615 | 5.65 | 3.72 | 4.43 |  | 1.972656 | 10 | 1.8801 | 7.32 | 4.56 | 5.92 |  |
|  |  | 7.921875 | 10 | 7.8332 | 2.33 | 0.81 | 2.20 |  | 7.890625 | 10 | 7.8773 | 2.98 | 2.73 | 1.51 |  |
|  |  | 31.6875 | 10 | 29.7218 | 3.58 | 0.00 | 3.58 |  | 31.5625 | 10 | 30.6690 | 6.55 | 0.00 | 6.55 |  |
|  |  | 126.75 | 10 | 127.4458 | 3.17 | 0.32 | 3.15 |  | 126.25 | 10 | 129.9423 | 5.70 | 2.60 | 5.15 |  |
|  |  | 507 | 10 | 533.9474 | 2.67 | 0.36 | 2.65 |  | 505 | 4 | 528.2789 | 8.67 | 0.00 | 8.67 |  |
| 1 | TNF-α | 0 | 5 | 0.0184 | 56.46 | 0.00 | 56.46 |  | 0 | 7 | 0.0330 | 86.54 | 69.58 | 54.22 |  |
|  |  |  |  |  |  |  |  |  | 0.018005 | 7 | 0.0314 | 53.55 | 59.04 | 19.64 |  |
|  |  | 0.077148 | 9 | 0.0858 | 31.21 | 0.00 | 31.21 |  | 0.072021 | 10 | 0.0845 | 47.64 | 23.89 | 41.97 |  |
|  |  | 0.308594 | 10 | 0.3271 | 10.99 | 0.00 | 10.99 |  | 0.288086 | 10 | 0.3277 | 11.66 | 0.00 | 11.66 |  |
|  |  | 1.234375 | 10 | 1.2409 | 3.96 | 2.80 | 2.95 |  | 1.152344 | 10 | 1.0833 | 5.55 | 4.17 | 3.93 |  |
|  |  | 4.9375 | 10 | 4.8523 | 5.28 | 2.20 | 4.85 |  | 4.609375 | 10 | 4.4672 | 4.01 | 0.00 | 4.01 |  |
|  |  | 19.75 | 10 | 19.0269 | 2.30 | 0.00 | 2.30 |  | 18.4375 | 10 | 17.6705 | 4.18 | 3.93 | 1.92 |  |
|  |  | 79 | 10 | 79.4273 | 2.99 | 2.59 | 1.73 |  | 73.75 | 10 | 77.0045 | 4.12 | 0.60 | 4.09 |  |
|  |  | 316 | 10 | 325.3935 | 3.10 | 0.00 | 3.10 |  | 295 | 4 | 316.3916 | 5.35 | 4.59 | 3.82 |  |
|  |  |  |  |  |  |  |  |  |  |  |  |  |  |  |  |
|  |  |  |  |  |  |  |  |  |  |  |  |  |  |  |  |
|  |  | **Batch 1** | | | | | |  | **Batch 2** | | | | | |  |
|  |  | **Defined** | **Total no.** | **Total** | **CV %** |  |  |  | **Defined** | **Total no.** | **Total** | **CV %** |  |  |  |
| **Panel** | **Marker** | **Conc.** | **of obs.** | **mean** | **All** | **Inter** | **Intra** |  | **Conc.** | **of obs.** | **mean** | **All** | **Inter** | **Intra** |  |
| 2 | IL12/23p40 | 0 | 4 | 0.2154 | 21.25 |  |  |  | 0 | 7 | 0.2898 | 55.82 | 37.79 | 41.52 |  |
|  |  |  |  |  |  |  |  |  | 0.17334 | 6 | 0.2245 | 80.27 | 0.00 | 80.27 |  |
|  |  | 0.664063 | 10 | 0.6936 | 29.13 | 0.00 | 29.13 |  | 0.693359 | 10 | 0.7335 | 25.94 | 0.00 | 25.94 |  |
|  |  | 2.65625 | 10 | 2.6939 | 6.33 | 3.69 | 5.29 |  | 2.773438 | 10 | 3.0866 | 16.77 | 0.00 | 16.77 |  |
|  |  | 10.625 | 10 | 10.4702 | 3.88 | 1.88 | 3.45 |  | 11.09375 | 10 | 10.8399 | 9.56 | 0.00 | 9.56 |  |
|  |  | 42.5 | 10 | 42.4503 | 7.33 | 2.97 | 6.77 |  | 44.375 | 10 | 42.6658 | 8.29 | 5.01 | 6.81 |  |
|  |  | 170 | 10 | 173.7552 | 5.36 | 0.00 | 5.36 |  | 177.5 | 10 | 179.9494 | 7.16 | 0.00 | 7.16 |  |
|  |  | 680 | 10 | 668.4294 | 5.03 | 0.00 | 5.03 |  | 710 | 10 | 727.6198 | 6.52 | 1.47 | 6.37 |  |
|  |  | 2720 | 10 | 2745.8135 | 2.85 | 0.00 | 2.85 |  | 2840 | 4 | 2815.9241 | 3.05 | 0.00 | 3.05 |  |
| 2 | IL-15 | 0 | 3 | 0.0155 | 96.55 |  |  |  | 0 | 5 | 0.0504 | 66.76 | 66.43 | 6.64 |  |
|  |  |  |  |  |  |  |  |  | 0.041077 | 9 | 0.0396 | 71.65 | 72.08 | 43.83 |  |
|  |  | 0.159668 | 10 | 0.1734 | 12.59 | 8.97 | 9.32 |  | 0.164307 | 10 | 0.1655 | 17.21 | 13.02 | 12.05 |  |
|  |  | 0.638672 | 10 | 0.6588 | 9.36 | 0.00 | 9.36 |  | 0.657227 | 10 | 0.7051 | 10.44 | 0.00 | 10.44 |  |
|  |  | 2.554688 | 10 | 2.6044 | 11.47 | 7.51 | 9.02 |  | 2.628906 | 10 | 2.6141 | 6.03 | 0.00 | 6.03 |  |
|  |  | 10.21875 | 10 | 10.0399 | 6.37 | 0.00 | 6.37 |  | 10.51563 | 10 | 10.2727 | 5.80 | 0.00 | 5.80 |  |
|  |  | 40.875 | 10 | 38.9558 | 5.36 | 0.00 | 5.36 |  | 42.0625 | 10 | 41.7077 | 7.31 | 0.00 | 7.31 |  |
|  |  | 163.5 | 10 | 152.8482 | 5.63 | 0.00 | 5.63 |  | 168.25 | 10 | 165.1912 | 5.13 | 0.00 | 5.13 |  |
|  |  | 654 | 10 | 740.4425 | 3.18 | 1.03 | 3.02 |  | 673 | 4 | 738.4192 | 4.62 | 0.38 | 4.61 |  |
| 2 | IL-5 | 0 | 5 | 0.0323 | 40.93 | 40.73 | 4.07 |  | 0 | 6 | 0.0315 | 60.11 | 56.18 | 23.45 |  |
|  |  |  |  |  |  |  |  |  | 0.053894 | 9 | 0.0574 | 46.91 | 23.78 | 41.00 |  |
|  |  | 0.19043 | 10 | 0.1970 | 18.81 | 0.00 | 18.81 |  | 0.215576 | 10 | 0.2253 | 13.90 | 0.00 | 13.90 |  |
|  |  | 0.761719 | 10 | 0.7372 | 8.88 | 5.16 | 7.43 |  | 0.862305 | 10 | 0.8849 | 8.30 | 1.03 | 8.24 |  |
|  |  | 3.046875 | 10 | 3.2920 | 29.54 | 0.00 | 29.54 |  | 3.449219 | 10 | 3.3166 | 6.71 | 5.30 | 4.47 |  |
|  |  | 12.1875 | 10 | 12.0845 | 6.47 | 3.16 | 5.74 |  | 13.79688 | 10 | 13.5140 | 2.69 | 0.61 | 2.63 |  |
|  |  | 48.75 | 10 | 48.4538 | 6.64 | 0.00 | 6.64 |  | 55.1875 | 10 | 56.1452 | 2.53 | 0.95 | 2.37 |  |
|  |  | 195 | 10 | 196.3171 | 5.37 | 0.00 | 5.37 |  | 220.75 | 10 | 225.4791 | 4.74 | 2.95 | 3.84 |  |
|  |  | 780 | 10 | 779.6123 | 7.86 | 0.00 | 7.86 |  | 883 | 4 | 880.1463 | 1.43 | 1.39 | 0.87 |  |
| 2 | IL-7 | 0 | 3 | 0.1003 | 6.22 |  |  |  | 0 | 7 | 0.0857 | 100.32 | 0.00 | 100.32 |  |
|  |  |  |  |  |  |  |  |  | 0.039063 | 7 | 0.0658 | 55.00 | 48.70 | 28.64 |  |
|  |  | 0.155518 | 10 | 0.1733 | 58.98 | 0.00 | 58.98 |  | 0.15625 | 10 | 0.1398 | 45.91 | 28.19 | 37.43 |  |
|  |  | 0.62207 | 10 | 0.6894 | 17.05 | 0.00 | 17.05 |  | 0.625 | 10 | 0.6772 | 11.96 | 0.00 | 11.96 |  |
|  |  | 2.488281 | 10 | 2.5893 | 7.24 | 3.19 | 6.58 |  | 2.5 | 10 | 2.4926 | 11.83 | 0.00 | 11.83 |  |
|  |  | 9.953125 | 10 | 9.3864 | 6.54 | 0.00 | 6.54 |  | 10 | 10 | 10.0231 | 3.20 | 1.79 | 2.72 |  |
|  |  | 39.8125 | 10 | 38.7343 | 2.89 | 1.57 | 2.48 |  | 40 | 10 | 39.8763 | 2.63 | 0.00 | 2.63 |  |
|  |  | 159.25 | 10 | 151.2590 | 4.92 | 0.00 | 4.92 |  | 160 | 10 | 159.2217 | 7.22 | 2.47 | 6.84 |  |
|  |  | 637 | 10 | 703.8015 | 5.18 | 0.00 | 5.18 |  | 640 | 4 | 651.1320 | 5.02 | 0.00 | 5.02 |  |
|  |  |  |  |  |  |  |  |  |  |  |  |  |  |  |  |
|  |  |  |  |  |  |  |  |  |  |  |  |  |  |  |  |
|  |  | **Batch 1** | | | | | |  | **Batch 2** | | | | | |  |
|  |  | **Defined** | **Total no.** | **Total** | **CV %** |  |  |  | **Defined** | **Total no.** | **Total** | **CV %** |  |  |  |
| **Panel** | **Marker** | **Conc.** | **of obs.** | **mean** | **All** | **Inter** | **Intra** |  | **Conc.** | **of obs.** | **mean** | **All** | **Inter** | **Intra** |  |
| 3 | IP-10 | 0 | 4 | 0.0185 | 50.70 |  |  |  | 0 | 6 | 0.2223 | 211.11 | 230.33 | 5.40 |  |
|  |  |  |  |  |  |  |  |  | 0.161743 | 9 | 0.1421 | 31.28 | 0.00 | 31.28 |  |
|  |  | 0.581054 | 10 | 0.6149 | 9.86 | 0.00 | 9.86 |  | 0.646972 | 10 | 0.6949 | 12.44 | 0.00 | 12.44 |  |
|  |  | 2.324219 | 10 | 2.3206 | 4.21 | 0.00 | 4.21 |  | 2.587891 | 10 | 2.6577 | 10.09 | 0.00 | 10.09 |  |
|  |  | 9.296875 | 10 | 8.9595 | 4.78 | 3.26 | 3.66 |  | 10.35156 | 10 | 10.5334 | 7.23 | 0.00 | 7.23 |  |
|  |  | 37.1875 | 10 | 36.9267 | 3.88 | 0.00 | 3.88 |  | 41.40625 | 10 | 40.7503 | 5.67 | 0.00 | 5.67 |  |
|  |  | 148.75 | 10 | 145.8248 | 5.34 | 1.43 | 5.16 |  | 165.625 | 10 | 160.3604 | 6.46 | 0.00 | 6.46 |  |
|  |  | 595 | 10 | 668.7228 | 2.62 | 1.00 | 2.45 |  | 662.5 | 10 | 683.5564 | 4.28 | 0.00 | 4.28 |  |
|  |  | 2380 | 10 | 2215.1922 | 9.64 | 0.00 | 9.64 |  | 2650 | 4 | 2633.8956 | 4.83 | 0.00 | 4.83 |  |
| 3 | MCP-1 | 0 | 5 | 0.1883 | 124.89 | 85.33 | 89.54 |  | 0 | 4 | 0.0446 | 94.17 |  |  |  |
|  |  |  |  |  |  |  |  |  | 0.029785 | 7 | 0.0313 | 78.08 | 75.64 | 36.59 |  |
|  |  | 0.114990 | 8 | 0.1609 | 73.16 | 0.00 | 73.16 |  | 0.119141 | 10 | 0.1373 | 40.61 | 0.00 | 40.61 |  |
|  |  | 0.459961 | 10 | 0.5008 | 10.03 | 4.03 | 9.28 |  | 0.476563 | 10 | 0.5185 | 9.53 | 0.00 | 9.53 |  |
|  |  | 1.839844 | 10 | 1.8257 | 7.60 | 0.00 | 7.60 |  | 1.90625 | 10 | 1.9295 | 7.33 | 4.73 | 5.81 |  |
|  |  | 7.359375 | 10 | 7.1548 | 4.26 | 0.00 | 4.26 |  | 7.625 | 10 | 7.1578 | 6.65 | 0.00 | 6.65 |  |
|  |  | 29.4375 | 10 | 29.0170 | 4.79 | 0.00 | 4.79 |  | 30.5 | 10 | 29.4570 | 7.06 | 0.00 | 7.06 |  |
|  |  | 117.75 | 10 | 120.8327 | 3.70 | 1.56 | 3.40 |  | 122 | 10 | 129.4223 | 5.19 | 2.31 | 4.71 |  |
|  |  | 471 | 10 | 474.3772 | 12.86 | 0.00 | 12.86 |  | 488 | 4 | 499.3594 | 5.03 | 0.00 | 5.03 |  |
| 3 | MCP-4 | 0 | 8 | 0.5891 | 67.48 | 17.34 | 65.44 |  | 0 | 5 | 1.5230 | 32.83 | 36.01 | 3.41 |  |
|  |  |  |  |  |  |  |  |  | 0.039368 | 4 | 1.1965 | 31.32 |  |  |  |
|  |  | 0.143555 | 8 | 0.8256 | 53.64 | 0.00 | 53.64 |  | 0.157471 | 4 | 1.3737 | 50.64 | 50.39 | 5.04 |  |
|  |  | 0.574219 | 6 | 0.6652 | 53.10 | 0.00 | 53.10 |  | 0.629883 | 6 | 1.3883 | 36.04 | 40.13 | 1.70 |  |
|  |  | 2.296875 | 10 | 2.1725 | 11.27 | 9.48 | 6.86 |  | 2.519531 | 10 | 2.7031 | 27.28 | 0.00 | 27.28 |  |
|  |  | 9.1875 | 10 | 9.5080 | 5.17 | 4.24 | 3.27 |  | 10.07813 | 10 | 9.8634 | 5.83 | 4.82 | 3.64 |  |
|  |  | 36.75 | 10 | 38.2608 | 3.48 | 3.34 | 1.48 |  | 40.3125 | 10 | 40.7664 | 5.44 | 0.00 | 5.44 |  |
|  |  | 147 | 10 | 141.6235 | 3.32 | 3.19 | 1.41 |  | 161.25 | 10 | 161.3649 | 3.80 | 0.00 | 3.80 |  |
|  |  | 588 | 10 | 597.7543 | 3.34 | 0.00 | 3.34 |  | 645 | 4 | 644.2681 | 1.40 | 0.00 | 1.40 |  |
| 3 | MDC | 0 | 1 | 2.8637 |  |  |  |  | 0 | 3 | 4.3533 | 115.25 |  |  |  |
|  |  |  |  |  |  |  |  |  | 0.616455 | 2 | 2.9598 | 61.39 |  |  |  |
|  |  | 2.319336 | 9 | 3.1104 | 38.43 | 39.16 | 19.15 |  | 2.46582 | 10 | 4.2464 | 57.16 | 14.09 | 55.60 |  |
|  |  | 9.277344 | 10 | 12.4744 | 11.62 | 5.09 | 10.58 |  | 9.863281 | 10 | 13.9067 | 13.28 | 12.12 | 6.76 |  |
|  |  | 37.10938 | 10 | 38.3830 | 1.77 | 1.23 | 1.35 |  | 39.45313 | 10 | 41.7041 | 3.55 | 0.00 | 3.55 |  |
|  |  | 148.4375 | 10 | 135.4589 | 2.09 | 1.83 | 1.18 |  | 157.8125 | 10 | 144.1231 | 3.37 | 0.00 | 3.37 |  |
|  |  | 593.75 | 10 | 590.5694 | 2.26 | 0.59 | 2.19 |  | 631.25 | 10 | 612.3447 | 4.45 | 0.00 | 4.45 |  |
|  |  | 2375 | 10 | 2603.6376 | 3.21 | 0.00 | 3.21 |  | 2525 | 10 | 2806.9107 | 6.23 | 3.39 | 5.35 |  |
|  |  | 9500 | 10 | 9258.7153 | 2.34 | 0.00 | 2.34 |  | 10100 | 4 | 9798.6796 | 4.12 | 0.00 | 4.12 |  |
| 3 | MIP-1α | 0 | 5 | 0.9145 | 81.50 | 0.00 | 81.50 |  | 0 | 6 | 3.7370 | 109.20 | 0.00 | 109.20 |  |
|  |  |  |  |  |  |  |  |  | 0.063477 | 2 | 1.5737 | 38.01 | 37.82 | 3.78 |  |
|  |  | 0.239014 | 7 | 1.1304 | 55.51 | 47.52 | 34.13 |  | 0.253906 | 2 | 2.9849 | 87.21 | 86.78 | 8.68 |  |
|  |  | 0.956055 | 7 | 1.1474 | 53.02 | 58.17 | 8.58 |  | 1.015625 | 8 | 2.1298 | 39.63 | 23.19 | 32.36 |  |
|  |  | 3.824219 | 10 | 3.7523 | 12.65 | 0.00 | 12.65 |  | 4.0625 | 10 | 4.3548 | 11.97 | 6.58 | 10.24 |  |
|  |  | 15.29688 | 10 | 15.1422 | 2.12 | 0.00 | 2.12 |  | 16.25 | 10 | 16.0811 | 5.27 | 0.00 | 5.27 |  |
|  |  | 61.1875 | 10 | 62.5387 | 3.76 | 0.00 | 3.76 |  | 65 | 10 | 65.1883 | 4.57 | 0.00 | 4.57 |  |
|  |  | 244.75 | 10 | 241.5392 | 2.79 | 0.00 | 2.79 |  | 260 | 10 | 259.8308 | 2.57 | 0.00 | 2.57 |  |
|  |  | 979 | 10 | 985.5965 | 5.57 | 0.00 | 5.57 |  | 1040 | 4 | 1044.9823 | 9.78 | 0.00 | 9.78 |  |
| 3 | MIP-1β | 0 | 6 | 0.5741 | 65.10 | 0.00 | 65.10 |  | 0 | 6 | 2.7513 | 196.68 | 0.00 | 196.68 |  |
|  |  |  |  |  |  |  |  |  | 0.064087 | 3 | 0.9927 | 74.66 |  |  |  |
|  |  | 0.249023 | 6 | 0.8635 | 88.95 | 84.00 | 50.90 |  | 0.256348 | 6 | 1.1362 | 45.36 | 37.35 | 26.27 |  |
|  |  | 0.996094 | 9 | 1.0241 | 39.83 | 32.80 | 25.70 |  | 1.025391 | 9 | 1.1363 | 39.55 | 0.00 | 39.55 |  |
|  |  | 3.984375 | 10 | 3.8252 | 12.06 | 0.00 | 12.06 |  | 4.101563 | 10 | 4.2312 | 10.10 | 0.00 | 10.10 |  |
|  |  | 15.9375 | 10 | 16.3614 | 5.71 | 1.49 | 5.54 |  | 16.40625 | 10 | 16.1153 | 4.73 | 0.00 | 4.73 |  |
|  |  | 63.75 | 10 | 64.7615 | 5.74 | 0.00 | 5.74 |  | 65.625 | 10 | 66.3127 | 3.25 | 0.00 | 3.25 |  |
|  |  | 255 | 10 | 250.6069 | 6.22 | 0.00 | 6.22 |  | 262.5 | 10 | 261.8827 | 2.73 | 0.00 | 2.73 |  |
|  |  | 1020 | 10 | 1038.6871 | 7.90 | 0.00 | 7.90 |  | 1050 | 4 | 1052.6674 | 1.32 | 0.00 | 1.32 |  |
| 3 | TARC | 0 | 5 | 0.5085 | 73.09 | 26.22 | 68.56 |  | 0 | 3 | 0.2753 | 18.38 | 18.29 | 1.83 |  |
|  |  |  |  |  |  |  |  |  | 0.089722 | 7 | 0.1295 | 75.38 | 0.00 | 75.38 |  |
|  |  | 0.349121 | 8 | 0.5010 | 44.10 | 0.00 | 44.10 |  | 0.358887 | 10 | 0.4479 | 29.14 | 14.12 | 25.92 |  |
|  |  | 1.396484 | 10 | 1.3244 | 27.83 | 7.47 | 26.93 |  | 1.435547 | 10 | 1.4256 | 8.44 | 0.00 | 8.44 |  |
|  |  | 5.585938 | 10 | 5.8417 | 6.22 | 3.54 | 5.25 |  | 5.742188 | 10 | 5.7252 | 7.48 | 0.00 | 7.48 |  |
|  |  | 22.34375 | 10 | 21.4048 | 7.46 | 0.00 | 7.46 |  | 22.96875 | 10 | 22.7109 | 4.69 | 0.00 | 4.69 |  |
|  |  | 89.375 | 10 | 90.7419 | 3.78 | 2.53 | 2.93 |  | 91.875 | 10 | 90.8122 | 8.33 | 0.00 | 8.33 |  |
|  |  | 357.5 | 10 | 362.7419 | 7.08 | 0.00 | 7.08 |  | 367.5 | 10 | 376.6362 | 5.62 | 0.00 | 5.62 |  |
|  |  | 1430 | 10 | 1428.1797 | 11.35 | 0.00 | 11.35 |  | 1470 | 4 | 1460.5200 | 5.42 | 0.00 | 5.42 |  |
|  |  |  |  |  |  |  |  |  |  |  |  |  |  |  |  |
|  |  |  |  |  |  |  |  |  |  |  |  |  |  |  |  |
|  |  | **Batch 1** | | | | | |  | **Batch 2** | | | | | |  |
|  |  | **Defined** | **Total no.** | **Total** | **CV %** |  |  |  | **Defined** | **Total no.** | **Total** | **CV %** |  |  |  |
| **Panel** | **Marker** | **Conc.** | **of obs.** | **mean** | **All** | **Inter** | **Intra** |  | **Conc.** | **of obs.** | **mean** | **All** | **Inter** | **Intra** |  |
| 4 | Tie-2 | 0 | 6 | 7.7697 | 60.74 | 39.93 | 46.76 |  | 0 | 4 | 2.5345 | 116.75 | 116.17 | 11.62 |  |
|  |  |  |  |  |  |  |  |  | 4.821777 | 2 | 9.6038 | 43.35 |  |  |  |
|  |  | 19.28711 | 10 | 19.0866 | 29.36 | 0.00 | 29.36 |  | 19.28711 | 10 | 21.8439 | 25.16 | 0.00 | 25.16 |  |
|  |  | 77.14844 | 10 | 72.3008 | 11.91 | 0.00 | 11.91 |  | 77.14844 | 10 | 79.2589 | 8.98 | 0.00 | 8.98 |  |
|  |  | 308.5938 | 10 | 309.6045 | 3.00 | 2.25 | 2.12 |  | 308.5938 | 10 | 306.5369 | 3.00 | 1.53 | 2.63 |  |
|  |  | 1234.375 | 10 | 1268.0143 | 2.67 | 0.00 | 2.67 |  | 1234.375 | 10 | 1234.7000 | 3.28 | 2.69 | 2.07 |  |
|  |  | 4937.5 | 10 | 4940.5754 | 2.21 | 0.00 | 2.21 |  | 4937.5 | 10 | 4834.1494 | 1.73 | 1.18 | 1.33 |  |
|  |  | 19750 | 10 | 19332.5770 | 1.83 | 0.00 | 1.83 |  | 19750 | 10 | 19624.4863 | 2.99 | 0.83 | 2.89 |  |
|  |  | 79000 | 10 | 79701.3696 | 1.55 | 0.00 | 1.55 |  | 79000 | 10 | 80802.4597 | 3.91 | 0.00 | 3.91 |  |
| 4 | VEGF-D | 0 | 4 | 0.8704 | 129.56 | 128.92 | 12.89 |  | 0 | 4 | 0.5468 | 165.60 | 164.78 | 16.48 |  |
|  |  |  |  |  |  |  |  |  | 1.394653 | 3 | 2.5886 | 46.20 | 49.97 | 21.68 |  |
|  |  | 5.578613 | 10 | 6.2813 | 18.46 | 15.40 | 11.39 |  | 5.578613 | 10 | 6.4307 | 22.98 | 8.38 | 21.58 |  |
|  |  | 22.31445 | 10 | 23.1550 | 5.92 | 0.00 | 5.92 |  | 22.31445 | 10 | 23.0902 | 7.81 | 0.00 | 7.81 |  |
|  |  | 89.25781 | 10 | 89.2027 | 4.60 | 0.00 | 4.60 |  | 89.25781 | 10 | 88.7958 | 4.13 | 0.00 | 4.13 |  |
|  |  | 357.0312 | 10 | 341.4937 | 2.30 | 0.64 | 2.22 |  | 357.0312 | 10 | 337.4449 | 2.46 | 0.00 | 2.46 |  |
|  |  | 1428.125 | 10 | 1432.4251 | 1.94 | 0.00 | 1.94 |  | 1428.125 | 10 | 1421.0803 | 3.62 | 1.18 | 3.44 |  |
|  |  | 5712.5 | 10 | 5922.7521 | 3.22 | 0.70 | 3.15 |  | 5712.5 | 10 | 6074.2118 | 5.69 | 1.81 | 5.43 |  |
|  |  | 22850 | 10 | 22655.5768 | 2.31 | 0.00 | 2.31 |  | 22850 | 10 | 22605.1892 | 3.22 | 0.00 | 3.22 |  |
|  |  |  |  |  |  |  |  |  |  |  |  |  |  |  |  |
|  |  |  |  |  |  |  |  |  |  |  |  |  |  |  |  |
|  |  | **Batch 1** | | | | | |  | **Batch 2** | | | | | |  |
|  |  | **Defined** | **Total no.** | **Total** | **CV %** |  |  |  | **Defined** | **Total no.** | **Total** | **CV %** |  |  |  |
| **Panel** | **Marker** | **Conc.** | **of obs.** | **mean** | **All** | **Inter** | **Intra** |  | **Conc.** | **of obs.** | **mean** | **All** | **Inter** | **Intra** |  |
| 5 | CRP | 0 | 5 | 0.4512 | 34.03 | 33.86 | 3.39 |  | 0 | 4 | 0.7151 | 58.04 | 57.75 | 5.78 |  |
|  |  |  |  |  |  |  |  |  | 2.336 | 4 | 3.0417 | 19.26 | 0.00 | 19.26 |  |
|  |  | 13.856 | 10 | 14.3031 | 4.67 | 0.00 | 4.67 |  | 11.68 | 10 | 11.9534 | 11.06 | 0.00 | 11.06 |  |
|  |  | 69.28 | 10 | 69.9528 | 3.24 | 0.00 | 3.24 |  | 58.4 | 10 | 57.1343 | 5.52 | 0.00 | 5.52 |  |
|  |  | 346.4 | 10 | 342.4467 | 5.43 | 2.14 | 5.04 |  | 292 | 10 | 289.6133 | 4.71 | 0.00 | 4.71 |  |
|  |  | 1732 | 10 | 1676.8310 | 2.17 | 0.85 | 2.01 |  | 1460 | 10 | 1439.2560 | 4.32 | 0.00 | 4.32 |  |
|  |  | 8660 | 10 | 8474.4473 | 2.08 | 2.00 | 0.89 |  | 7300 | 10 | 7345.2757 | 4.48 | 0.00 | 4.48 |  |
|  |  | 43300 | 10 | 49690.9443 | 4.35 | 0.00 | 4.35 |  | 36500 | 10 | 38033.4447 | 4.71 | 2.27 | 4.20 |  |
|  |  | 216500 | 10 | 194126.2926 | 6.66 | 0.00 | 6.66 |  | 182500 | 10 | 177357.2516 | 6.60 | 0.00 | 6.60 |  |
| 5 | SAA | 0 | 4 | 4.4061 | 46.72 | 46.49 | 4.65 |  | 0 | 2 | 5.9259 | 119.91 | 119.31 | 11.93 |  |
|  |  |  |  |  |  |  |  |  | 2.656 | 2 | 19.6778 | 70.12 | 69.78 | 6.98 |  |
|  |  | 17.088 | 10 | 19.5309 | 45.23 | 0.00 | 45.23 |  | 13.28 | 9 | 19.1862 | 81.41 | 100.83 | 26.79 |  |
|  |  | 85.44 | 10 | 101.3887 | 7.70 | 0.00 | 7.70 |  | 66.4 | 10 | 79.0922 | 23.26 | 13.50 | 19.47 |  |
|  |  | 427.2 | 10 | 461.2013 | 4.03 | 2.43 | 3.32 |  | 332 | 10 | 370.5498 | 9.18 | 0.00 | 9.18 |  |
|  |  | 2136 | 10 | 1982.3883 | 3.01 | 0.00 | 3.01 |  | 1660 | 10 | 1608.2356 | 6.07 | 0.00 | 6.07 |  |
|  |  | 10680 | 10 | 9603.3201 | 3.36 | 2.11 | 2.72 |  | 8300 | 10 | 7427.5804 | 6.39 | 0.00 | 6.39 |  |
|  |  | 53400 | 10 | 55272.6868 | 4.71 | 0.10 | 4.71 |  | 41500 | 10 | 38972.0050 | 4.35 | 0.00 | 4.35 |  |
|  |  | 267000 | 10 | 292506.9965 | 5.60 | 3.90 | 4.22 |  | 207500 | 10 | 251874.7173 | 6.34 | 4.59 | 4.63 |  |
| 5 | sICAM-1 | 0 | 5 | 0.3533 | 44.62 | 44.40 | 4.44 |  | 0 | 5 | 0.3705 | 97.53 | 96.37 | 17.59 |  |
|  |  |  |  |  |  |  |  |  | 0.58816 | 3 | 1.0743 | 66.76 | 60.43 | 44.98 |  |
|  |  | 3.616 | 10 | 3.6137 | 17.96 | 8.14 | 16.23 |  | 2.9408 | 10 | 3.0038 | 26.52 | 0.00 | 26.52 |  |
|  |  | 18.08 | 10 | 18.6604 | 6.85 | 3.60 | 5.95 |  | 14.704 | 10 | 14.2527 | 8.40 | 0.00 | 8.40 |  |
|  |  | 90.4 | 10 | 90.5785 | 5.87 | 1.88 | 5.60 |  | 73.52 | 10 | 74.4120 | 8.12 | 0.00 | 8.12 |  |
|  |  | 452 | 10 | 445.9408 | 5.18 | 2.76 | 4.48 |  | 367.6 | 10 | 377.6107 | 7.99 | 2.31 | 7.69 |  |
|  |  | 2260 | 10 | 2227.7999 | 5.02 | 4.28 | 2.99 |  | 1838 | 10 | 1847.8389 | 6.16 | 0.00 | 6.16 |  |
|  |  | 11300 | 10 | 11670.7174 | 5.11 | 3.67 | 3.75 |  | 9190 | 10 | 8989.2171 | 4.58 | 0.00 | 4.58 |  |
|  |  | 56500 | 10 | 56235.9469 | 5.17 | 0.00 | 5.17 |  | 45950 | 10 | 46391.8250 | 5.70 | 0.00 | 5.70 |  |
| 5 | sVCAM1 | 0 | 5 | 0.9401 | 63.65 | 0.00 | 63.65 |  | 0 | 4 | 2.9972 | 121.64 |  |  |  |
|  |  |  |  |  |  |  |  |  | 0.62912 | 3 | 3.5741 | 39.00 | 0.00 | 39.00 |  |
|  |  | 3.424 | 9 | 4.9867 | 58.50 | 37.41 | 46.43 |  | 3.1456 | 8 | 7.7876 | 184.93 | 0.00 | 184.93 |  |
|  |  | 17.12 | 10 | 20.6665 | 12.09 | 0.00 | 12.09 |  | 15.728 | 10 | 17.1781 | 11.19 | 0.00 | 11.19 |  |
|  |  | 85.6 | 10 | 86.8492 | 4.85 | 0.00 | 4.85 |  | 78.64 | 10 | 80.7223 | 5.08 | 0.00 | 5.08 |  |
|  |  | 428 | 10 | 386.7806 | 3.42 | 0.00 | 3.42 |  | 393.2 | 10 | 369.3929 | 6.20 | 3.13 | 5.45 |  |
|  |  | 2140 | 10 | 2112.4444 | 4.09 | 1.98 | 3.64 |  | 1966 | 10 | 1944.3459 | 5.23 | 0.00 | 5.23 |  |
|  |  | 10700 | 10 | 12057.5114 | 6.14 | 5.80 | 2.81 |  | 9830 | 10 | 10463.0705 | 5.01 | 1.34 | 4.85 |  |
|  |  | 53500 | 10 | 52336.1127 | 4.16 | 0.00 | 4.16 |  | 49150 | 10 | 48534.5633 | 2.90 | 0.00 | 2.90 |  |
|  |  |  |  |  |  |  |  |  |  |  |  |  |  |  |  |
