# Supplementary material for: Immunological profile in cerebrospinal fluid of patients with multiple sclerosis after treatment switch to rituximab and compared with healthy controls
Source: PLoS One. 2018 Feb 8;13(2):e0192516. doi: 10.1371/journal.pone.0192516 (PMC5805315; doi:10.1371/journal.pone.0192516)
Supplement: S3 Table — All values in pg/mL. Differences at various time-points versus HC reaching statistical significance after correction for 42 multiple comparisons according to Holm-Bonferroni are indicated in bold. **N = number of samples accepted for statistical analysis. (DOCX) [file pone.0192516.s003.docx]

| **Analyte** | **HC** | **MS Month 0** | | | **MS Month 12** | | | **MS month 24** | | | **Comment** |
| --- | --- | --- | --- | --- | --- | --- | --- | --- | --- | --- | --- |
|  |  | **Median** | **p-value*** | **N**** | **Median** | **p-value*** | **N**** | **Median** | **p-value*** | **N**** |  |
| IP-10 (CXCL10) | 284 | 938 | **<0.0001** | (55 vs 66) | 616 | **<0.0001** | (55 vs 68) | 597 | **<0.0001** | (55 vs 68) | Statistically significant difference versus HC at all time points |
| IL-12/IL-23p40 | 3.77 | 7.23 | **<0.0001** | (52 vs 62) | 5.19 | **0.0012** | (52 vs 60) | 6.29 | **0.0012** | (52 vs 60) |  |
| s-VCAM-1 | 6150 | 8070 | **<0.0001** | (54 vs 70) | 7350 | **0.0011** | (54 vs 69) | 7580 | **0.0011** | (54 vs 69) |  |
| IL-8 (CXCL8) | 34.1 | 44.0 | **0.0000** | (55 vs 69) | 41.7 | **0.0000** | (55 vs 69) | 42.1 | **0.0000** | (55 vs 69) |  |
| MIP-1β  (CCL4) | 10.5 | 15.5 | **0.0000** | (48 vs 56) | 14.2 | **0.0001** | (48 vs 58) | 14.1 | **0.0001** | (48 vs 58) |  |
| CRP | 1360 | 2450 | **0.0007** | (53 vs 66) | 2240 | **0.0001** | (53 vs 67) | 2290 | **0.0001** | (53 vs 67) |  |
| IL-15 | 2.27 | 2.49 | **0.0020** | (55 vs 70) | 2.28 | 0.2967 | (55 vs 69) | 2.42 | 0.2967 | (55 vs 69) | No statistically significant difference versus HC after therapy switch |
| s-ICAM-1 | 1630 | 1980 | **0.0008** | (54 vs 70) | 1830 | 0.0274 | (54 vs 69) | 1980 | 0.0274 | (54 vs 69) |  |
| SAA | 667 | 944 | **0.0002** | (50 vs 67) | 814 | 0.0108 | (50 vs 64) | 906 | 0.0108 | (50 vs 64) |  |
| IL-7 | 1.35 | 1.10 | **0.0026** | (54 vs 48) | 1.22 | 0.1300 | (54 vs 56) | 1.13 | 0.1300 | (54 vs 56) |  |
| IL-6 | 1.11 | 1.42 | 0.0118 | (49 vs 59) | 1.21 | 0.4938 | (49 vs 55) | 1.23 | 0.4938 | (49 vs 55) | No statistically significant difference versus HC at any timepoint. |
| VEGFD | 41.0 | 41.4 | 0.6907 | (55 vs 70) | 44.6 | 0.2198 | (55 vs 67) | 45.8 | 0.1808 | (55 vs 59) |  |
| IL-5 | 0.57 | 0.51 | 0.0110 | (53 vs 60) | 0.52 | 0.0236 | (53 vs 57) | 0.53 | 0.0236 | (53 vs 57) |  |
| MCP-1 (CCL2) | 317 | 327 | 0.2482 | (55 vs 68) | 316 | 0.9838 | (55 vs 68) | 316 | 0.5306 | (55 vs 59) |  |
