# Supplementary material for: Immunological profile in cerebrospinal fluid of patients with multiple sclerosis after treatment switch to rituximab and compared with healthy controls
Source: PLoS One. 2018 Feb 8;13(2):e0192516. doi: 10.1371/journal.pone.0192516 (PMC5805315; doi:10.1371/journal.pone.0192516)
Supplement: S1 Fig — All values in pg/mL. The fence of the whiskers represents the max-min values. The green lines represent the LLoQ, the blue lines represent the min-max of the HC. (PDF) [file pone.0192516.s004.pdf]

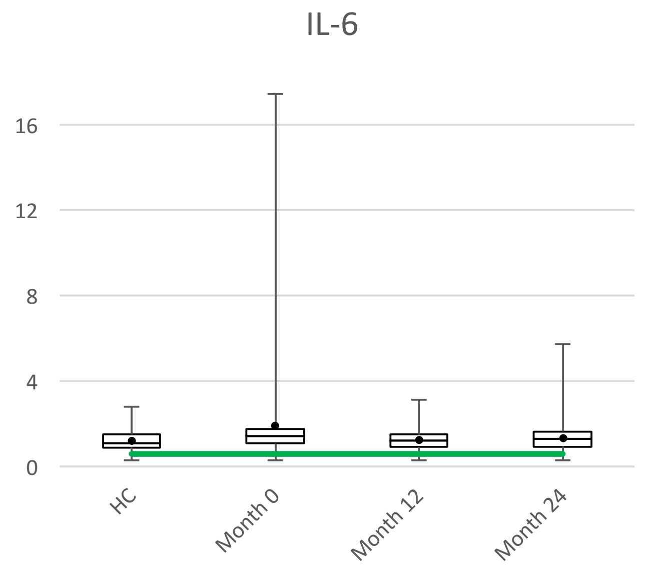
IL-6
16
12
8
4
0
HC
Month 0
Month 12
Month 24

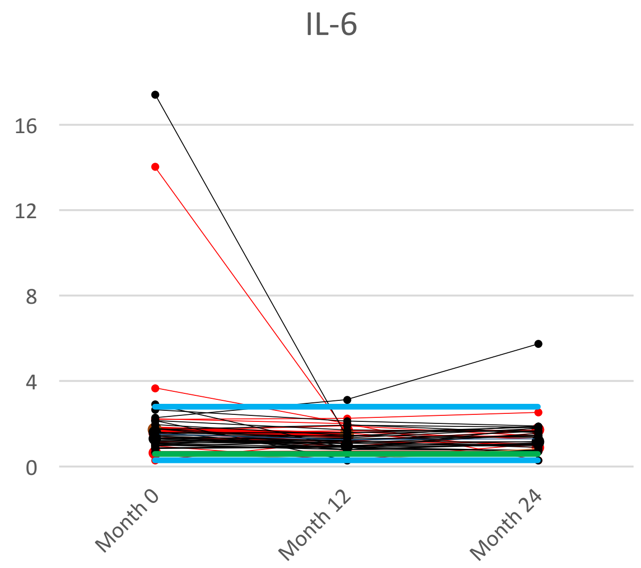
IL-6
16
12
8
4
0
Month 0
Month 12
Month 24

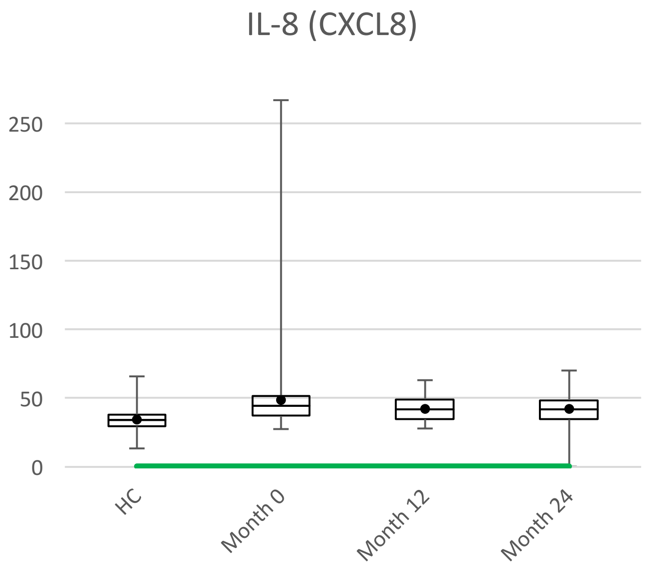
IL-8 (CXCL8)
250
200
150
100
50
0
HC
Month 0
Month 12
Month 24

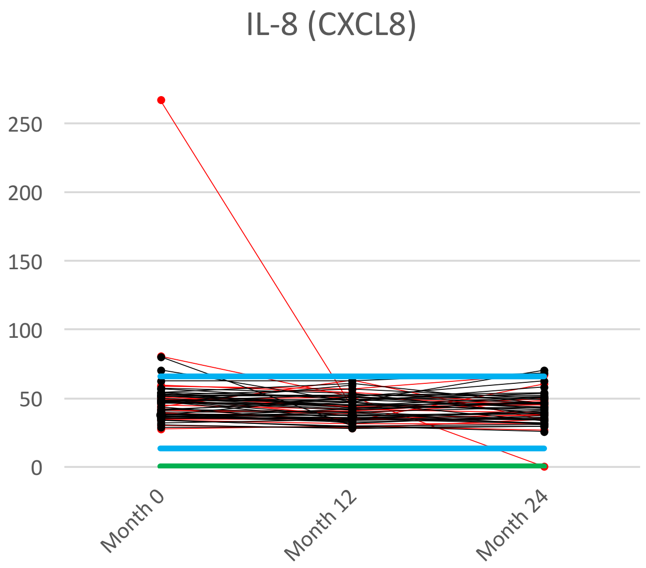
IL-8 (CXCL8)
250
200
150
100
50
0
Month 0
Month 12
Month 24

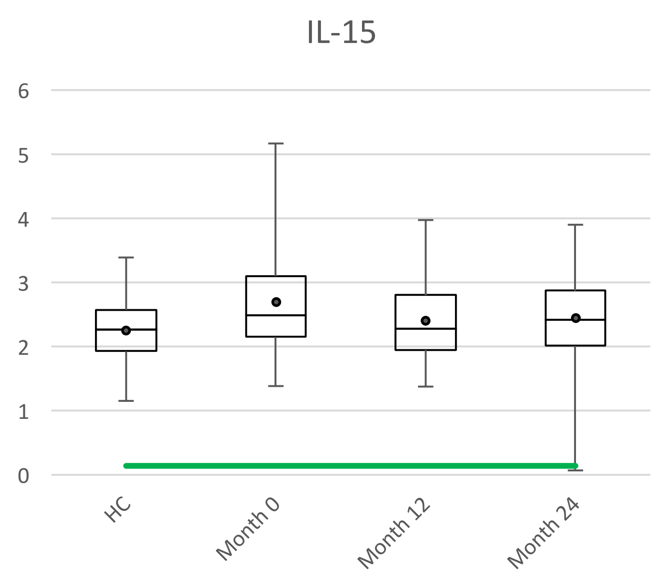
IL-15
6
5
4
3
2
1
0
HC
Month 0
Month 12
Month 24

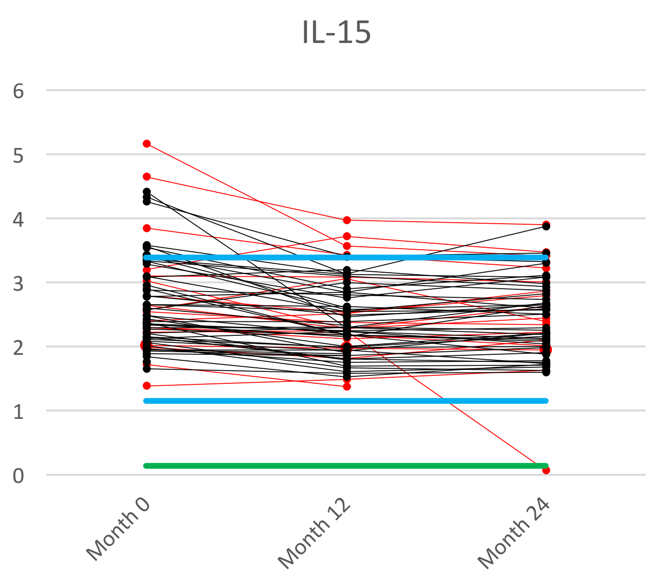
IL-15
6
5
4
3
2
1
0
Month 0
Month 12
Month 24

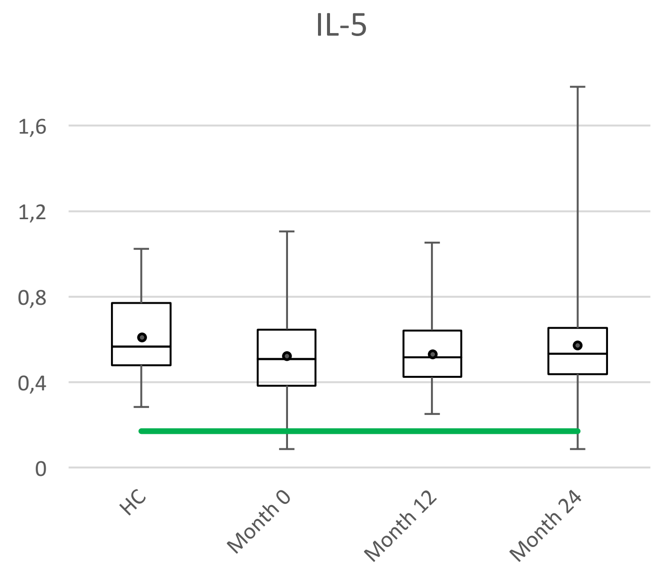
IL-5
1,6
1,2
0,8
0,4
0
HC
Month 0
Month 12
Month 24

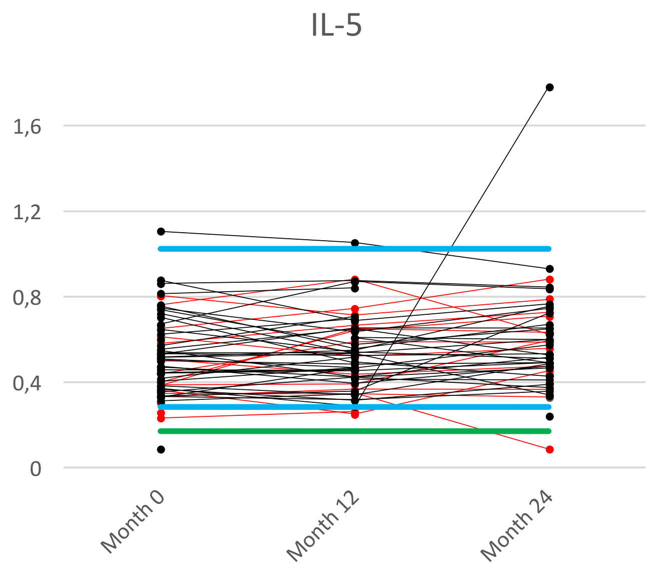
IL-5
1,6
1,2
0,8
0,4
0
Month 0
Month 12
Month 24

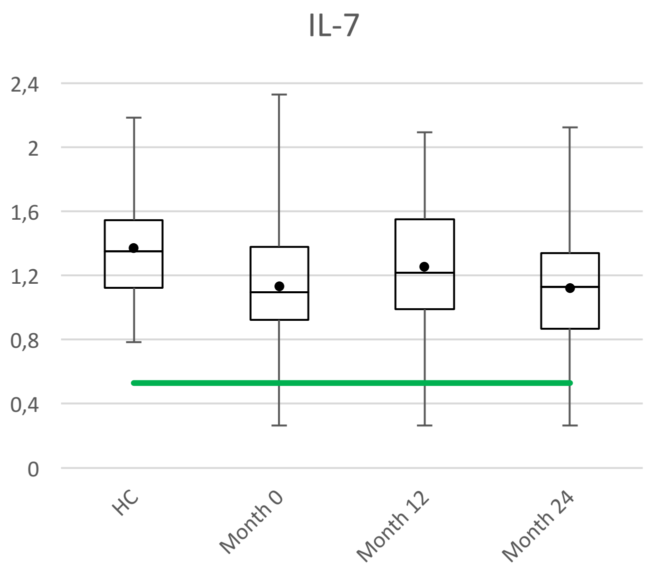
IL-7
2,4
2
1,6
1,2
0,8
0,4
0
HC
Month 0
Month 12
Month 24

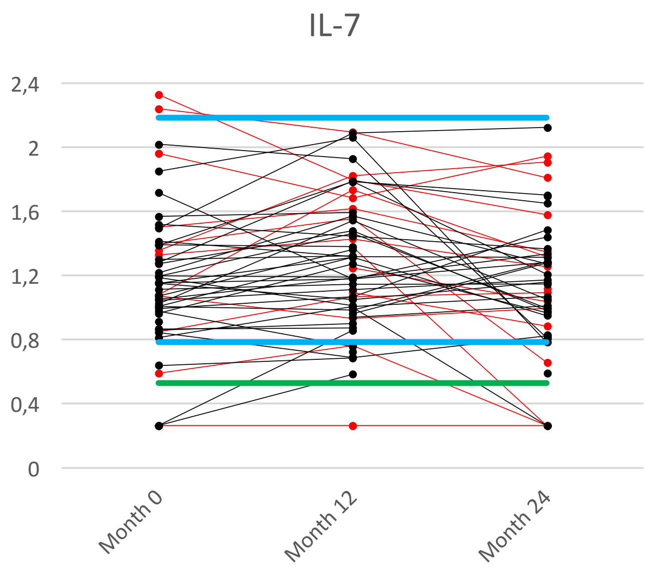
IL-7
2,4
2
1,6
1,2
0,8
0,4
0
Month 0
Month 12
Month 24

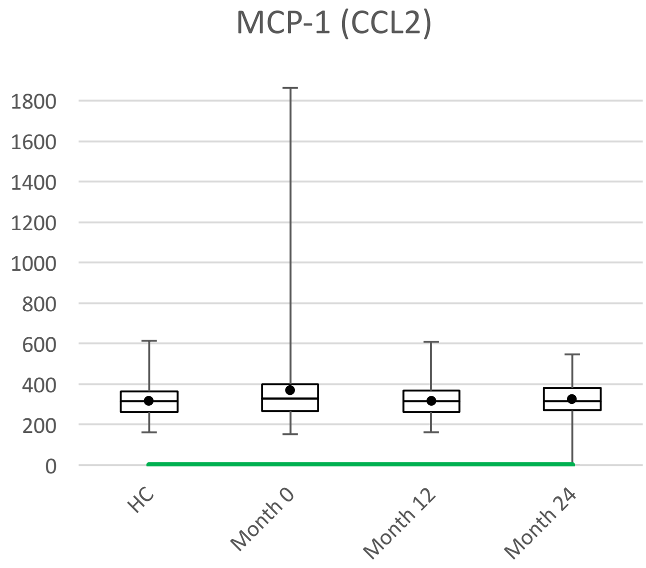
MCP-1 (CCL2)
1800
1600
1400
1200
1000
800
600
400
200
0
HC
Month 0
Month 12
Month 24

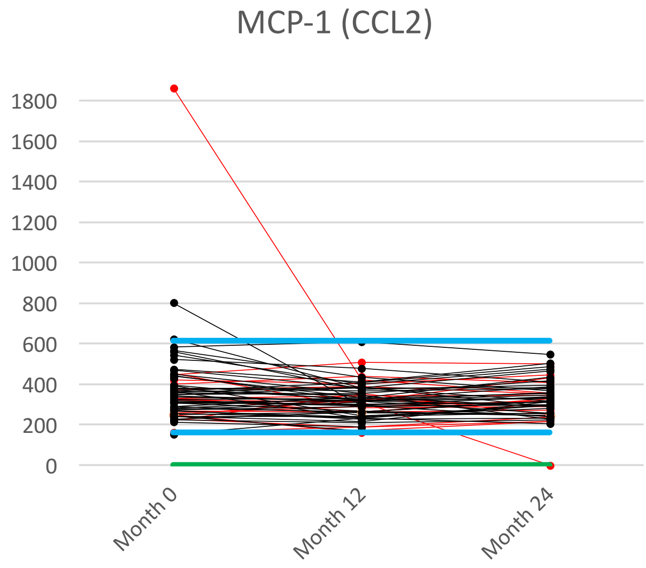
MCP-1 (CCL2)
1800
1600
1400
1200
1000
800
600
400
200
0
Month 0
Month 12
Month 24

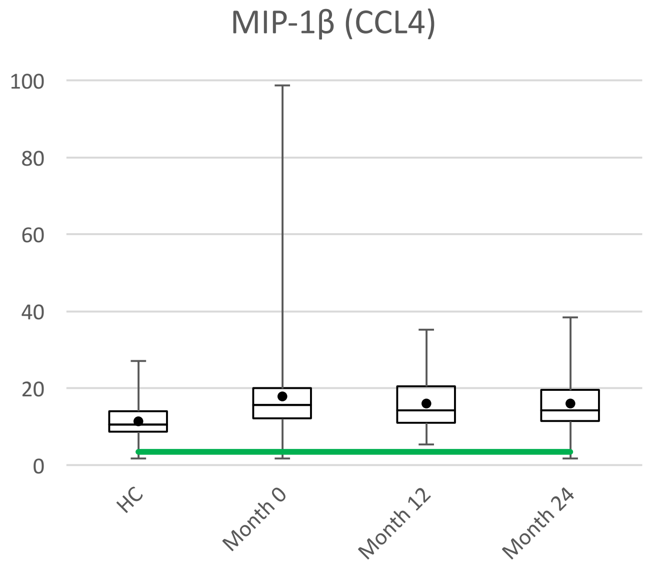
MIP-1β (CCL4)
100
80
60
40
20
0
HC
Month 0
Month 12
Month 24

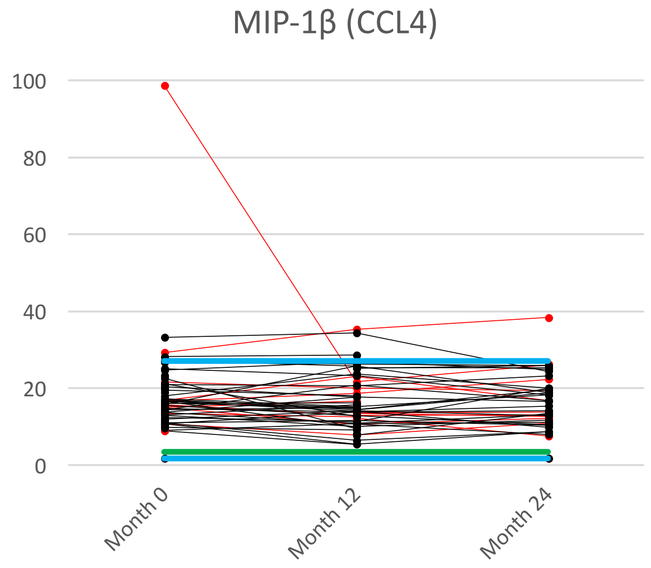
MIP-1β (CCL4)
100
80
60
40
20
0
Month 0
Month 12
Month 24

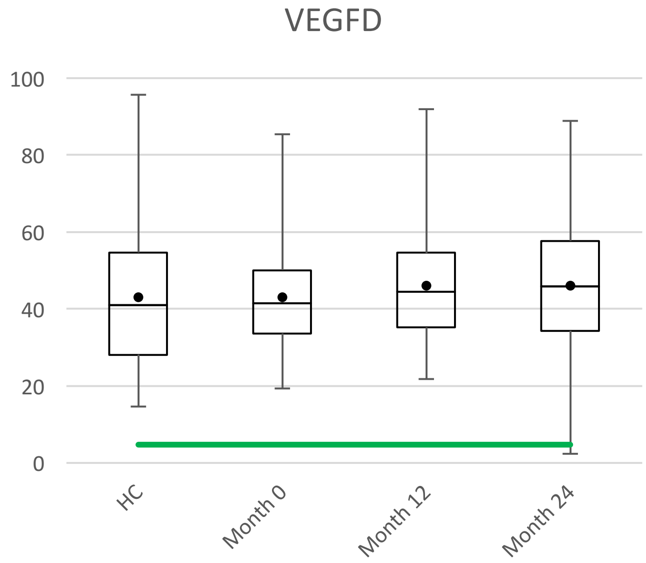
VEGFD
100
80
60
40
20
0
HC
Month 0
Month 12
Month 24

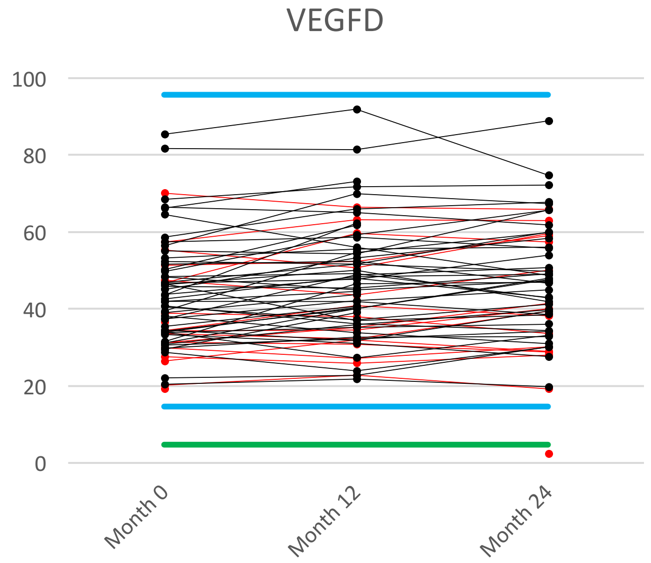
VEGFD
100
80
60
40
20
0
Month 0
Month 12
Month 24

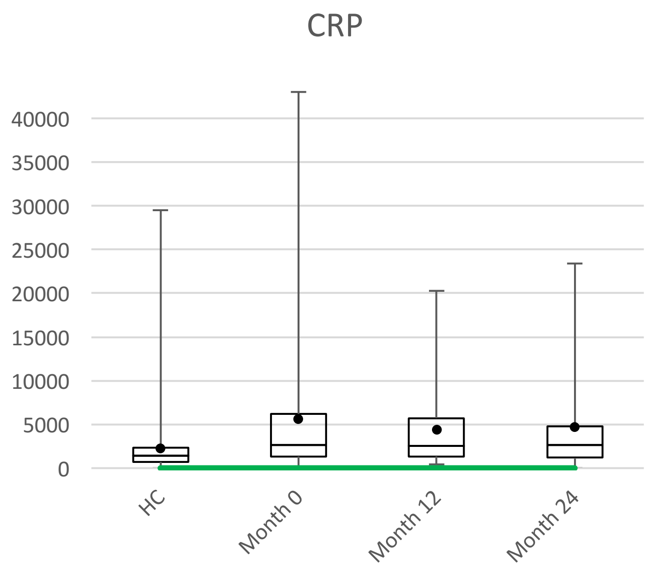
CRP
40000
35000
30000
25000
20000
15000
10000
5000
0
HC
Month 0
Month 12
Month 24

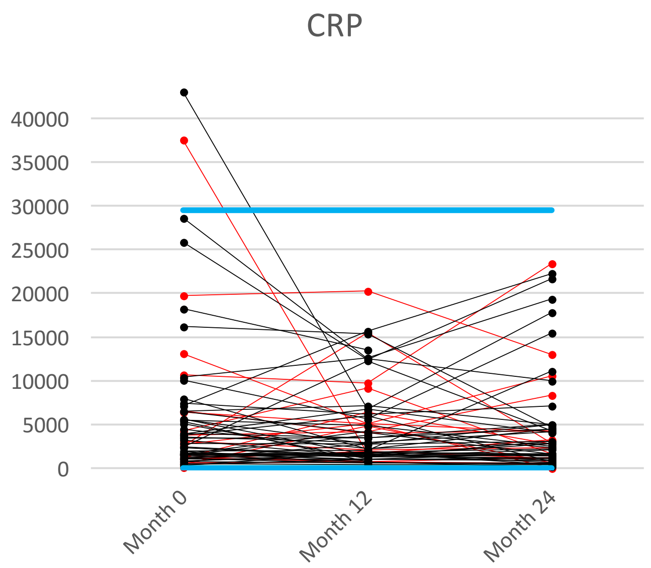
CRP
40000
35000
30000
25000
20000
15000
10000
5000
0
Month 0
Month 12
Month 24

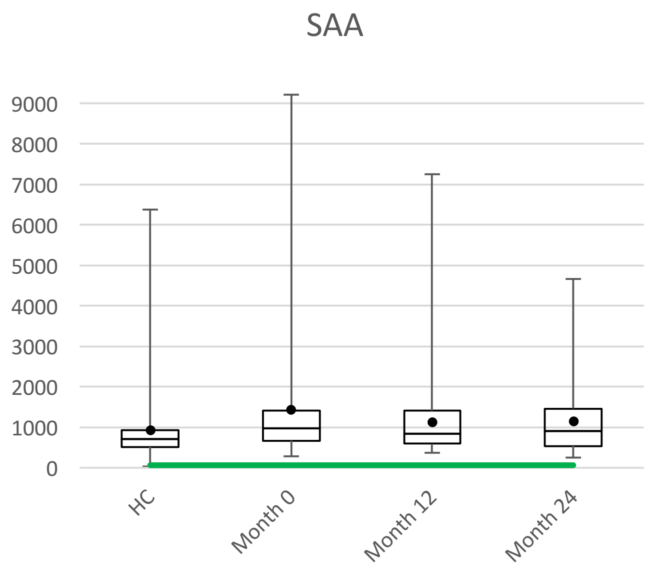
SAA
9000
8000
7000
6000
5000
4000
3000
2000
1000
0
HC
Month 0
Month 12
Month 24

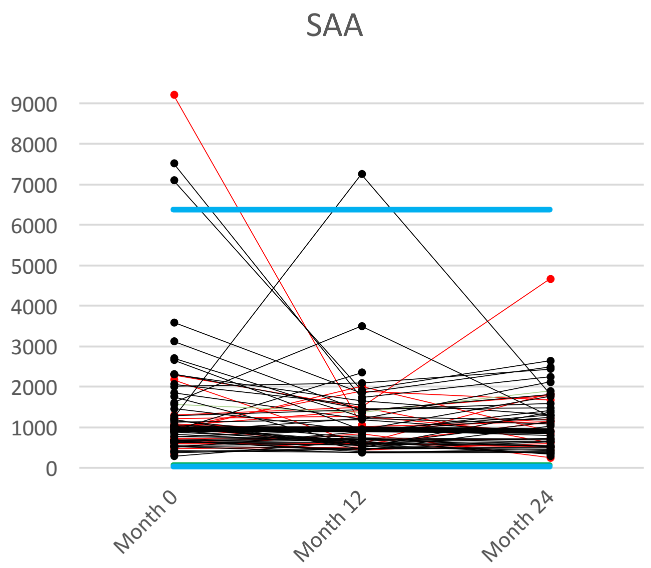
SAA
9000
8000
7000
6000
5000
4000
3000
2000
1000
0
Month 0
Month 12
Month 24

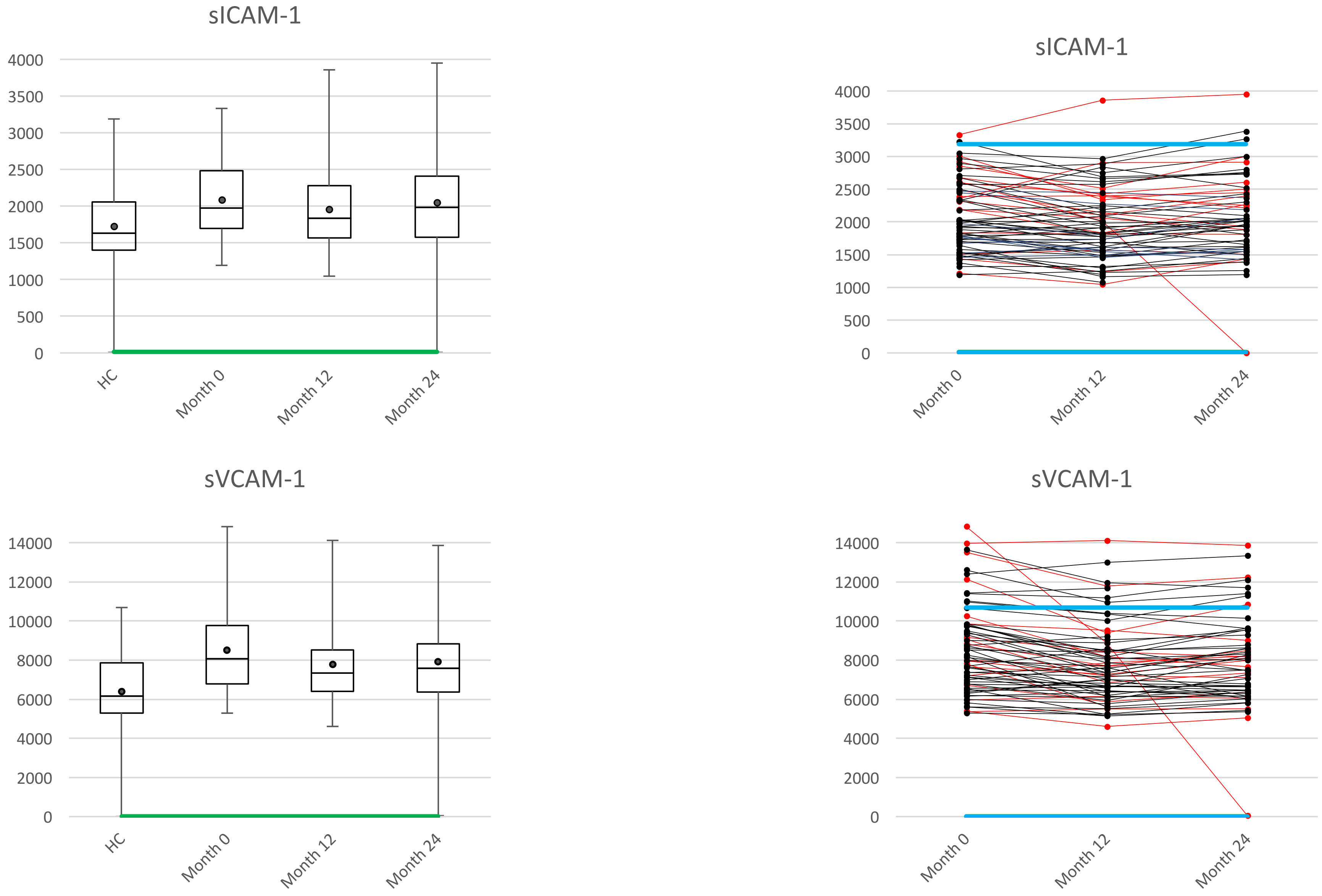

S3 Fig. Boxplots and dot-line diagrams for the analytes not presented in the main manuscript. All values in pg/mL. The fence of the whiskers respresent the max-min values. Green line represent the LLOQ, blue lines represents the min-max of the HC.
